# Supplementary material for: A harmonized resource of integrated prostate cancer clinical, -omic, and signature features
Source: Sci Data. 2023 Jul 5;10:430. doi: 10.1038/s41597-023-02335-4 (PMC10322899; doi:10.1038/s41597-023-02335-4)
Supplement: Supplementary file 1 — Supplementary Figures [file 41597_2023_2335_MOESM1_ESM.docx]

Supplementary Figures for

A harmonized resource of integrated prostate cancer clinical, -omic, and signature features

Teemu D. Laajala^1,2^, Varsha Sreekanth^2^, Alex Soupir^3^, Jordan Creed^3^, Anni S. Halkola^1^, Federico C.F. Calboli^1,4^, Kalaimathy Singaravelu^1^, Michael V. Orman^2^, Christelle Colin-Leitzinger^3^, Travis Gerke^5^, Brooke L. Fridley^3,*^, Svitlana Tyekucheva^6,*^, James C. Costello^2,7,*^

^1^Department of Mathematics and Statistics, University of Turku, Turku, Finland

^2^Department of Pharmacology, University of Colorado Anschutz Medical Campus, Aurora, CO, USA

^3^Department of Biostatistics and Bioinformatics, Moffitt Cancer Center, Tampa, FL, USA

^4^Natural Resources Institute Finland (Luke), F-31600, Jokioinen, Finland

^5^Department of Cancer Epidemiology, Moffitt Cancer Center, Tampa, FL, USA

^6^Department of Data Science, Dana-Farber Cancer Institute; Department of Biostatistics, Harvard T.H. Chan School of Public Health, Boston, MA, USA

^7^University of Colorado Cancer Center, University of Colorado Anschutz Medical Campus, Aurora, CO, USA

*These authors jointly supervised this work

Corresponding authors: Teemu D Laajala ([teelaa@utu.fi](mailto:teelaa@utu.fi)), Svitlana Tyekucheva ([svitlana@jimmy.harvard.edu](mailto:svitlana@jimmy.harvard.edu)), and James C Costello ([james.costello@cuanschutz.edu](mailto:james.costello@cuanschutz.edu))

**Supplementary Figures**

**Figure S1**: **Workflow of the *curatedPCaData* MultiAssayExperiment-object generation.** **a**) Primary raw data is extracted mainly using the GEOquery and cBioPortalData packages. Raw data are processed according to latest annotations with the help of biomaRt and assay-specific packages, and then processed using affy, oligo, limma, and rCGH packages where appropriate; **b**) Key data compoents are stored via the *ExperimentHub* web storage service and downloaded or stored locally. From *ExperimentHub*, MAE-object is constructed while providing access to the primary data (GEX, CNA, and MUT), offering derived variables (decompositions and scores), and corresponding clinical metadata (MAE colData-slot)

**Figure S2**: **Impact of Gene Missingness on Risk and AR Score Reliability**. Prostate risk scores and AR score were benchmarked using datasets from the *curatedPCaData* package to determine how missing genes impacted their reliability. The number of trials are listed at the bottom of each figure panel. **a**) TCGA was used to assess Oncotype DX risk score removing between 1 and 5 genes. **b**) Kunderfranco et al. was used to assess Prolaris risk score by removing between 1 and 10 genes. TCGA was leveraged to assess gene removal for **c**) Decipher (1-5 genes) and **d**) Androgen Receptor (1-10 genes).
